# Double‐level osteotomy (DLO) for varus deformity results in over a decade of mean survival without compromising subsequent total knee arthroplasty function at a mean 26‐year follow‐up

**DOI:** 10.1002/jeo2.70140

**Published:** 2025-01-08

**Authors:** Sean C. Clark, Karissa N. Simon, Daniel B. F. Saris, Michael J. Taunton, Aaron J. Krych, Mario Hevesi

**Affiliations:** ^1^ Department of Orthopedic Surgery Mayo Clinic Rochester Minnesota USA; ^2^ Department of Orthopedic Surgery, University Medical Center Utrecht University Utrecht The Netherlands

**Keywords:** distal femoral osteotomy, DLO, double‐level osteotomy, high tibial osteotomy, total knee arthroplasty

## Abstract

**Purpose:**

Double‐level osteotomies (DLOs) have shown promising results for knee joint preservation, however, most ultimately progress in terms of degenerative disease resulting in conversion to total knee arthroplasty (TKA). Therefore, the purpose of this study was to examine the time to TKA conversion, long‐term clinical outcomes and revision rates of patients who have undergone TKA after prior ipsilateral DLO.

**Methods:**

Patients who underwent simultaneous or staged DLO and subsequently underwent conversion to TKA at a single academic institution from 1997 to 2022 were evaluated. The type of osteotomy performed (opening‐ vs. closing‐wedge), osteotomy hardware fixation, when and if osteotomy hardware was removed, implanted TKA components and revision rates were recorded. Postoperative outcomes, including Forgotten Joint Score‐12 (FJS‐12), Tegner Activity Scale score and subjective knee preference were also obtained.

**Results:**

A total of 22 patients (24 knees) underwent TKA following DLO and were followed for an average of 26.1 ± 7.7 years. The average time from DLO to TKA conversion was 14.1 ± 6.5 years, with 70.8% of knees converting to TKA more than 10 years after DLO. The mean follow‐up after conversion to TKA was 12.0 ± 7.7 years. Only 12.5% of patients received a varus‐valgus or hinged‐constrained TKA. At the final follow‐up, the mean FJS‐12 was 75.8 ± 23.1, while the mean Tegner Activity Scale score was 2.5 ± 1.1. Seventy‐seven percent of patients had no subjective knee preference or preferred their DLO‐TKA knee. Only two knees (7.4%) underwent subsequent revision after index arthroplasty at a mean of 9.3 years postoperatively.

**Conclusions:**

A majority of DLOs (70.8%) converted to TKA after more than a decade. Subsequent TKA function was favorable as most patients had either no subjective knee preference or preferred their DLO‐TKA knee. This study demonstrates both long‐term joint preservation and uncompromised TKA function after prior DLO.

**Level of Evidence:**

Level IV.

**Abbreviations**
DFO: distal femoral osteotomy
DLO: double‐level osteotomy
FJS‐12: Forgotten Joint Score‐12
HTO: high tibial osteotomy
TKA: total knee arthroplasty

## INTRODUCTION

Limb mechanical malalignment significantly impacts the distribution of loading forces on cartilage and subchondral bone within both the medial and lateral compartments of the knee, exacerbating the progression of osteoarthritis. Osteotomies performed around the knee address these issues and play a pivotal role in redistributing compartmental loads. This not only provides pain relief but also holds the potential to delay or even avoid the need for a total knee arthroplasty (TKA) [13, 28].

A varus malalignment is typically associated with a deformity of the tibia [7, 15, 20], while a valgus malalignment is generally associated with a deformity of the femur [9, 27]. However, a severe deformity may be a result of both tibial and femoral deformities [13, 18]. In the treatment of these cases, overcorrection of the coronal axis through a distal femoral osteotomy (DFO) or high tibial osteotomy (HTO) may lead to excess joint line obliquity and increase the loading forces on the articular surfaces [13]. Thus, combining a DFO and HTO to form a double‐level osteotomy (DLO) becomes imperative to maintain joint line obliquity and better preserve the native knee joint [6, 13, 30].

The concept of a DLO was initially introduced by Benjamin in 1969, primarily focusing on pain alleviation rather than biomechanical realignment [10, 11]. In the ensuing decades, outcomes have shown inconsistencies [23, 32], primarily attributed to inadequate preoperative planning and an insufficient emphasis on restoring the mechanical axis [16]. However, in the early 2000s, Babis et al. and Saragaglia et al. presented more promising and sustainable results by addressing these critical factors [10, 30]. Nevertheless, many patients are expected to progress in terms of degenerative disease and ultimately undergo a TKA [35]. This prompts surgeons to carefully weigh the advantages of joint‐preserving interventions, such as a DLO, against their potential implications on the outcomes of subsequent procedures.

The impact of a DLO on subsequent TKA function is currently unknown. Therefore, the purpose of this study was to examine the time to TKA conversion, long‐term clinical outcomes and revision rates of patients who have undergone TKA after prior ipsilateral DLO. We hypothesize that these patients will exhibit long‐term joint preservation, favorable TKA outcomes and low rates of revision surgeries.

## MATERIALS AND METHODS

**Figure 1:**
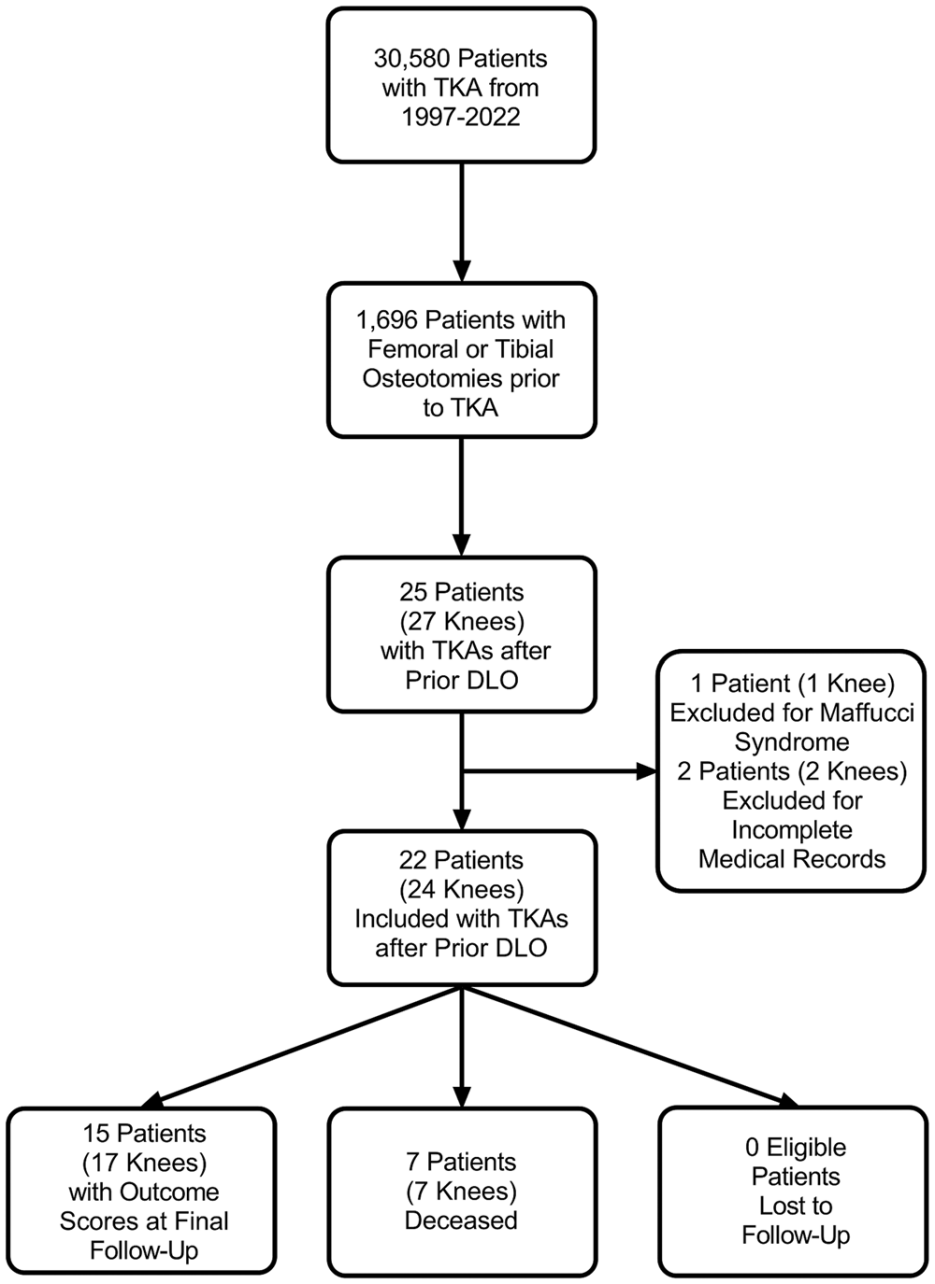
Patient screening and enrollment based on inclusion criteria.

All patients who underwent TKA after ipsilateral DLO at a single academic institution from 1997 to 2022 were reviewed following approval from our Institutional Review Board (IRB #15‐000601). Inclusion criteria consisted of patients who underwent simultaneous or staged DLO and subsequently underwent conversion to TKA of the ipsilateral knee. Three patients were excluded from the study, including one with Maffucci syndrome who had numerous operations on their DLO knee, which were likely to affect postoperative outcomes, as well as two who had incomplete medical records (Figure 1).

**Figure 2:**
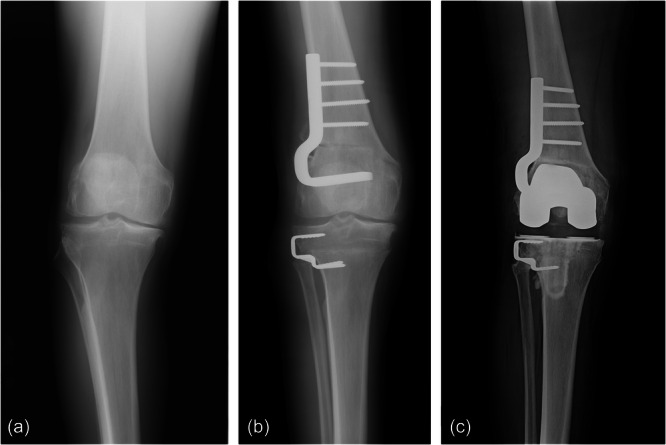
(a and b) Anteroposterior radiographs demonstrating varus knee deformity of the right knee treated with a double‐level osteotomy using a lateral closing‐wedge osteotomy with plate and screws for femoral fixation and a lateral closing‐wedge osteotomy with staples for tibial fixation. (c) Subsequently, after 19.2 years, the patient underwent total knee arthroplasty conversion with a posterior stabilized implant system.

Clinical and demographic data were obtained from the Mayo Clinic Total Joint Registry. This registry prospectively records demographics, operative data and postoperative complications [12]. A total of 22 patients (24 knees) underwent TKA following prior ipsilateral DLO (Figure 2). Patients underwent DLO from 1983 to 2019, with 83.3% (20/24) being performed at Mayo Clinic, 12.5% (3/24) being performed at outside institutions and 4.2% (1/24) with the DFO being performed at Mayo Clinic while the HTO was performed at an outside institution. All primary TKAs were performed at Mayo Clinic from 1997 to 2022, with indications for conversion being osteoarthritis progression or pain. The type of osteotomy performed (opening‐ vs. closing‐wedge), osteotomy hardware fixation (plate and screws vs. staples), when and if osteotomy hardware was removed, implanted TKA components (presence of femoral and tibial stems, implant constraint, polyethylene thickness) and revision rates were obtained. Postoperative outcomes, including Forgotten Joint Score‐12 (FJS‐12), Tegner Activity Scale score and subjective knee preference, were also obtained. Subjective knee preference was acquired by asking, ‘Which knee would you consider to be the better knee?’.

### Statistical analysis

Data was extracted with continuous variables being reported as mean ± standard deviation, while categorical variables were reported as frequencies with percentages. Kaplan–Meier curves were used to evaluate the probability of survival from DLO to TKA and from TKA to revision surgery.

## RESULTS

The overall mean follow‐up of the study (DLO to final follow‐up) was 26.1 ± 7.7 years (range 3.8–34.7 years). The mean age at the time of DLO was 45.5 ± 11.4 years, with most patients being male (81.8%, 18/22). All DLOs were performed for the correction of severe varus deformities. Lateral closing‐wedge osteotomies were performed 95.8% (23/24) of the time for both DFOs and HTOs. Plate and screws were always used for DFO fixation (100%, 24/24), while staples were used for the majority of HTOs (79.2%, 19/24) (Table 1). Three DLOs (12.5%, 3/24) were completed via staged procedures, while the rest were done simultaneously. Excluding hardware removal, no patients underwent revision after DLO.

**Table 1:** Patient demographics and DLO characteristics.

**Age**	
At DLO (years)	45.5 ± 11.4
At TKA following DLO (years)	59.6 ± 14.3
**Sex**	
Female	4 (18.2%)
Male	18 (81.8%)
**DLO laterality**	
Left	17 (70.8%)
Right	7 (29.2%)
**DFO osteotomy**	
Medial opening‐wedge	1 (4.2%)
Lateral closing‐wedge	23 (95.8%)
**DFO fixation**	
Plate and screws	24 (100%)
Staples	0 (0%)
**HTO osteotomy**	
Medial opening‐wedge	1 (4.2%)
Lateral closing‐wedge	23 (95.8%)
**HTO fixation**	
Plate and screws	6 (25.0%)
Staples	18 (75.0%)

Abbreviations: DFO, distal femoral osteotomy; DLO, double‐level osteotomy; HTO, high tibial osteotomy; TKA, total knee arthroplasty.

**Figure 3:**
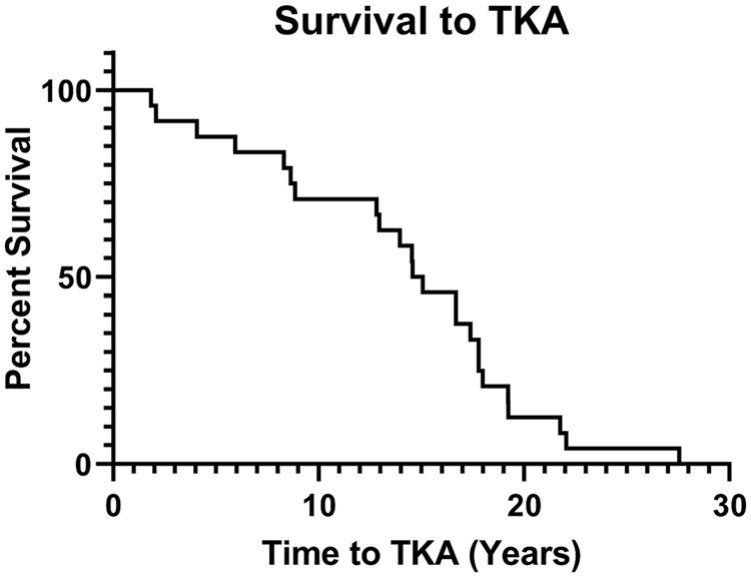
Kaplan–Meier curve showing the probability of survival from total knee arthroplasty (TKA) conversion after double‐level osteotomy.

The average time from DLO to conversion to TKA was 14.0 ± 6.5 years (range 1.8–27.6 years), with 70.8% (17/24) of knees converting to TKA more than 10 years after DLO (Figure 3). The average age at the time of TKA was 59.6 ± 14.3 years. Fifty‐four percent (13/24) of knees had DFO hardware removed at the time of TKA, while 75.0% (18/24) of HTO hardware was not removed. Forty‐six percent (11/24) of knees received a stemmed TKA femoral component, while 45.8% (11/24) received a stemmed tibial component. Eighty‐eight percent (21/24) of knees received a posterior‐stabilized implant, while 8.3% (2/24) received a varus‐valgus constraint, and 4.2% (1/24) received a hinge prosthesis. The average polyethylene insert was 11.3 ± 2.3 mm (Table 2). No conversions to TKA were due to DLO failure, and manual TKAs were performed on all knees.

**Table 2:** Hardware removal and components used at the time of DLO‐TKA.

**DFO hardware removal**	
Prior to TKA	2 (8.3%)
At the time of TKA	13 (54.2%)
No	9 (37.5%)
**HTO hardware removal**	
Prior to TKA	1 (4.2%)
At the time of TKA	5 (20.8%)
No	18 (75.0%)
**Stemmed femoral component**	
Yes	11 (45.8%)
No	13 (54.2%)
**Stemmed tibial component**	
Yes	11 (45.8%)
No	13 (54.2%)
**TKA implant constraint**	
Posterior‐stabilized	21 (87.5%)
Varus‐valgus	2 (8.3%)
Hinged	1 (4.2%)
**Polyethylene insert (mm)**	11.3 ± 2.3

Abbreviations: DFO, distal femoral osteotomy; DLO, double‐level osteotomy; HTO, high tibial osteotomy; TKA, total knee arthroplasty.

**Figure 4:**
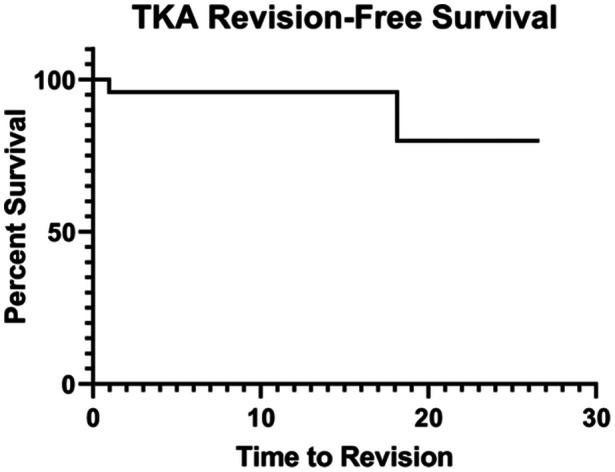
Kaplan–Meier curve showing the probability of survival from revision surgery after primary total knee arthroplasty (TKA).

The mean follow‐up after conversion to TKA was 12.0 ± 7.7 years (range 1.5–26.6 years). Seven patients were deceased at the time of the final follow‐up. Postoperative outcome scores were obtained on the remaining 15 patients (17 knees) who were eligible for follow‐up. The mean FJS‐12 was 75.8 ± 23.1, while the mean Tegner Activity Scale score was 2.5 ± 1.1. Of the 13 patients with unilateral DLOs, eight patients (61.5%) had no subjective knee preference, two (15.4%) preferred their DLO‐TKA knee and three (23.1%) preferred their non‐DLO only knee. Two patients (8.7%) underwent subsequent revision after their primary TKA: one at 18.1 years for implant loosening, while the other underwent irrigation and debridement at 6 months, a manipulation under anesthesia at 8 months and a conversion to a hinge prosthesis due to arthrofibrosis at 1.0 year (Figure 4).

## DISCUSSION

The most important findings of the present study were that DLOs that were performed for varus deformities resulted in a mean survival of almost 15 years prior to conversion to TKA. Additionally, DLO did not affect subsequent TKA function as patients had favorable FJS‐12 scores with over 75.0% having either no subjective knee preference or preferring their DLO‐TKA knee. Finally, after conversion to TKA, at a mean follow‐up of 12.0 years, only two knees (8.3%) underwent subsequent revision, none of which were for infection.

Our study has the longest reported mean follow‐up after DLO in the literature, with an average of 14.1 years prior to conversion to TKA. However, previous studies have also demonstrated favorable outcomes and proven success in joint preservation [8, 22]. Babis et al. published one of the earliest case series of patients who underwent DLO and found Knee Society Scores significantly improved pre‐ to postoperatively (*p* < 0.001) at a mean follow‐up of 6.9 years, with only 4.2% of patients undergoing subsequent TKA [10]. A separate study reported favorable clinical outcomes at a mid‐term follow‐up of 3.8 years with no patients converting to TKA [30]. Finally, Schuster et al. reported excellent short‐term outcomes at a mean follow‐up of 1.5 years, with all patients at the final follow‐up stating that they would retrospectively undergo the procedure again [33].

To our knowledge, the present study was the first to examine clinical outcomes of TKAs after prior DLO. The normative values for the FJS‐12 were established by Giesinger et al. by sampling 2000 participants that were representative of the United States population. The authors found the median normative FJS‐12 for knees to be 75.0 [19], which was effectively the same as our cohort, with a score of 75.8. Chalmers et al. studied a case series of 29 patients who underwent TKA after DFO. The authors found that Knee Society Scores significantly improved pre‐ to postoperatively, and the 10‐year survivorship was 88% [14]. Hevesi et al. performed a self‐matched cohort study with patients who underwent bilateral TKAs after prior unilateral HTO. The authors demonstrated no significant difference in outcomes between HTO‐TKA and TKA‐only knees, with 81% of patients having either no subjective knee preference or preference for the HTO‐TKA knee, similar to our current study. Additionally, most patients received posterior‐stabilized implants, with only 7% of the HTO‐TKA knees undergoing subsequent revision [21]. Similarly, in our study, 87.5% of patients received a posterior‐stabilized implant after DLO, and only 8.3% of patients underwent subsequent revision at a mean follow‐up of 12.0 years. It is important to note that almost half of the patients required either femoral or tibial stemmed components.

The indication for a DLO rather than a HTO is when the lateral distal femoral angle is > 90°, the medial proximal tibial angle (MPTA) < 84° or the planned correction of the MPTA is ≥ 95° [5, 26, 31]. Multiple studies have shown that knees with a predicted postoperative MPTA > 95° had worse clinical outcomes and increased joint line obliquity if treated with an HTO in comparison to a DLO [3, 4]. Abs et al. compared patients with MPTAs < 84°, who either underwent DLO or HTO and found that HTOs produced less physiologic joint line obliquity and had a greater incidence of hinge fractures [1]. However, Rosso et al. found that a postoperative MPTA ≥ 95° after HTO was not significantly associated with inferior clinical outcomes at a mean follow‐up of 10 years [29].

Additional studies have similarly reported DLO to be superior for joint preservation in comparison to HTO for patients with severe varus deformity. In patients with a predicted postoperative MPTA ≥ 95°, DLO demonstrated significantly decreased patellofemoral osteoarthritis in comparison to patients who underwent opening‐wedge HTO (*p* = 0.002) [2]. Similarly, after HTO, a joint line obliquity ≥6° was significantly associated with a smaller medial joint space width, while a joint line obliquity ≥5° resulted in excessive shear stress on the tibial articular cartilage [25, 34]. Nakayama et al. performed a second‐look arthroscopy at an average of 17.1 months after DLO and found cartilage repair to have occurred in over 90% of the medial femoral and tibial condyles [24].

Although a DLO is performed less frequently in the United States due to technical demand and being more invasive than HTOs, Feucht et al. found that in patients with varus deformities with ≥3° tibiofemoral angles, only 12% of patients could be corrected via an HTO while 63% required a DLO, if anatomic correction of an MPTA ≤ 90° was desired. However, if overcorrection was accepted with an MPTA of ≤95°, then 57% of patients could be corrected with an HTO, with 33% still requiring a DLO [17]. Further randomized controlled studies are necessary to understand the indications and long‐term clinical outcomes of DLOs and HTOs for patients with severe varus deformities further. Nonetheless, this is the first study to demonstrate that patients can expect uncompromised TKA function and favorable outcomes after prior ipsilateral DLO.

The current study is not without limitations. While our study had an extended mean follow‐up of 26.1 years, we remain limited by the small sample size and the lack of a control group. However, DLOs are uncommon procedures, and for patients to subsequently undergo TKA with an extended follow‐up is extremely rare. Nevertheless, this study suggests that favorable DLO‐TKA outcomes are commonly observed for the life of the implant and the patient. Given the extended follow‐up, hard copy films for pre‐ and postoperative MPTAs could not be uniformly obtained to evaluate the inclination of the joint line. Our institution serves as a tertiary referral center, with approximately 2500 TKAs being performed annually by surgeons with substantial technical expertise for complex primary TKAs [21]. Thus, it is likely that well‐experienced surgeons played a vital role in the outcomes observed. On a related note, intraoperative decision‐making in the setting of potentially challenging anatomy, as well as assessment of bone quality for the use of femoral and tibial stems, was at the discretion of the individual surgeon. Finally, our results are susceptible to the biases inherent to retrospective reviews, which include not identifying all eligible patients and incomplete recordkeeping.

## CONCLUSIONS

In conclusion, a majority (70.8%) of DLOs converted to TKA after more than a decade. Subsequent TKA function was favorable as most patients had either no subjective knee preference or preferred their DLO‐TKA knee. This study demonstrates both long‐term joint preservation and uncompromised TKA function after prior DLO.

## AUTHOR CONTRIBUTIONS

**Sean C. Clark**: Data curation; formal analysis; methodology; writing—original draft. **Karissa N. Simon**: Data curation; formal analysis; writing—original draft. **Daniel B. F. Saris**: Conceptualization; supervision; writing—review and editing. **Michael J. Taunton**: Conceptualization; supervision; writing—review and editing. **Aaron J. Krych**: Conceptualization; supervision; writing—review and editing. **Mario Hevesi**: Conceptualization; project administration; supervision; writing—review and editing.

## CONFLICT OF INTEREST STATEMENT

The authors declare no conflicts of interest.

## ETHICS STATEMENT

This retrospective chart review study involving human participants was in accordance with the ethical standard of the Mayo Clinic Institutional Review Board (#15‐000601). Informed consent was obtained from all individual participants included in the study.

## Data Availability

Data that supports the findings of this study are available from the corresponding author upon reasonable request.
